# Antibody response of growing German Holstein bulls to a vaccination against bovine viral diarrhea virus (BVDV) is influenced by *Fusarium* toxin exposure in a non-linear fashion

**DOI:** 10.1007/s12550-018-0307-4

**Published:** 2018-02-07

**Authors:** Sven Dänicke, Janine Winkler, Ulrich Meyer, Susanne Kersten, Kerstin Wernike, Martin Beer, Jana Frahm

**Affiliations:** 1 Institute of Animal Nutrition, Friedrich-Loeffler-Institute (FLI), Federal Research Institute for Animal Health, Bundesallee 37, 38116 Braunschweig, Germany; 2 Institute of Diagnostic Virology, Friedrich-Loeffler-Institute (FLI), Federal Research Institute for Animal Health, Südufer 10-Insel Riems, 17493 Greifswald, Germany

**Keywords:** Growing bulls, Hematology, Clinical chemistry, Immune system, Mycotoxins, Deoxynivalenol, Zearalenone

## Abstract

The *Fusarium* toxin deoxynivalenol (DON) is a frequent contaminant of feedstuffs and is supposed to interfere with immune responses. As the relevance for growing bulls is less clear than for other livestock, the trial was designed according to the dose-response principal with a control group fed a diet with background contamination (CON, 0.36 mg DON per kilogram dry matter [DM]) and three groups with increasing concentrations of DON (mg/kg DM); FUS I, 3.01; FUS II, 5.66; FUS III, 8.31. Half of each treatment group was vaccinated against BVDV at days 1 and 21 of the 70 days lasting experiment. Sequential blood samples were collected for determination of antibody titers to BVDV and for hematological and clinical-chemical traits. Antibody response was strongest in group FUS II while group FUS III responded weakest. This group showed the lowest proportion of CD4+ T cells, but also the highest levels of liver lesion indicating enzyme activities in blood. BVDV-vaccination induced a pronounced decrease in red blood count indices, which occurred dose-dependently at a higher level in the FUS-fed groups. The obvious interactions between DON exposure and BVDV-vaccination require further elucidation.

**Electronic supplementary material:**

The online version of this article (10.1007/s12550-018-0307-4) contains supplementary material, which is available to authorized users.

## Introduction

Deoxynivalenol (DON) is a *Fusarium* toxin and a highly prevalent feed contaminant. It often co-occurs with zearalenone (ZEN) and an array of further *Fusarium* toxins but constitutes the major part of the toxicological potential of such multi-contaminated feedstuffs (Binder et al. 2007; Nesic et al. 2014). Cattle is regarded as quite resistant to DON due to its capability to degrade DON to the less toxic metabolite de-epoxy-DON (de-DON) pre-systemically as mediated by ruminal micro-organisms (Dänicke and Brezina 2013). Irrespective of this general detoxification capability, a guidance value for critical concentration of 5 mg DON per kilogram complete feeding stuff at a reference dry matter (DM) content of 88% for ruminants was established by the European Commission (European Commission 2006) based on literature evaluation performed by European Food Safety Authority (European Food Safety Authority 2004). Due to the limited data base, nearly exclusively performance parameters such as DM-intake and live weight gain were used as toxicological endpoints. On the other hand, on a molecular level, DON is known to inhibit protein synthesis and to trigger a so called ribotoxic stress response (Iordanov et al. 1997; Pestka et al. 2004; Pestka 2007) which is characterized by an activation of mitogen-activated protein kinases (MAPKs). These effects were particularly demonstrated for immune cells giving rise to conclude immuno-modulating effects of DON.

Bovine viral diarrhea virus (BVDV) is an enveloped single-stranded, positive sense RNA pestivirus of the family *Flaviviridae* that occurs in two genotypes (BVDV-1 and BVDV-2) and two biotypes (non-cytopathogenic [ncp] and cytopathogenic [cp]) (Brackenbury et al. 2003). BVDV is capable of infecting granulocytes, macrophages, antigen-presenting myeloid cells, CD4+ and CD8+, and T cells and B cells. Thus, it affects both innate and acquired immune responses (Chase et al. 2004). Infection with BVDV is highly prevalent worldwide in cattle, is characterized by a number of clinical manifestations, and is therefore subject to various prevention strategies, including vaccination (Lanyon et al. 2014).

Considering the possible interference of DON and its known and putative derivatives with immune cells, we hypothesized that exposure of bulls to DON modulates immune-related traits, including the antibody response to BVDV vaccination.

To verify this hypothesis, increasing dietary DON concentrations was tested either in unvaccinated or BVDV-vaccinated bulls. The results of the present paper complete the findings on general growth performance and toxin residues in physiological specimens as reported recently (Winkler et al. 2016).

## Materials and methods

### Experimental design, diets, and procedures

The experiment was performed at the experimental station of the Institute of Animal Nutrition Braunschweig. The study complied with the European Community regulations concerning the protection of experimental animals and was approved by the Lower Saxony State Office for Consumer Protection and Food Safety (LAVES), Germany.

The experiment was described in detail by Winkler et al. (2016). Briefly, the trial was designed according to the dose-response principal with a control group fed a diet with background contamination (CON, 0.08 mg ZEN and 0.36 mg DON per kilogram dry matter [DM]) and three groups with increasing concentrations of DON and ZEN; FUS I, 0.28 mg ZEN and 3.01 mg DON per kilogram DM; FUS II, 0.48 mg ZEN and 5.66 mg DON per kilogram DM; FUS III, 0.69 mg ZEN and 8.31 mg DON per kilogram DM. The increase in toxin concentrations of the total mixed rations (TMR) is the result of the exchange of background-contaminated concentrate feed with the *Fusarium* toxin-contaminated concentrate feed (Table 1). *Fusarium* toxin-contaminated maize grains and cobs were used as toxin sources. Grass and press pulp silage (47 and 20% on DM basis, respectively) served as roughage and completed the TMR.

**Table 1:** Components, energy content and composition of the concentrates (*n* = 2), grass silage, and pressed beet pulp silage (data from (Winkler et al. 2016))

	Concentrate			
	CON	FUS	Grass silage	Pressed beet pulp silage
Components [%]				
Rapeseed extraction meal	20.0	20.0		
Barley	30.3	29.0		
Maize	36.0			
*Fusarium* toxin contaminated maize		36.0		
*Fusarium* toxin contaminated maize cobs		1.3		
Dried beet pulp	9.5	9.5		
Soybean oil	1.2	1.2		
Urea	1.0	1.0		
Mineral and vitamin premix*	2.0	2.0		
DM^a^ [%]	86.7	86.9	30.2	28.4
Nutrient composition[g/kg DM]				
Crude ash	53	54	110	59
Crude protein	153	158	133	74
Crude fat	49	50	39	12
Crude fiber	75	78	277	197
Acid detergent fiber	103	107	299	241
Neutral detergent fiber	227	233	501	523
Mycotoxin composition [mg/kg DM]				
ZEN	0.01	1.95	0.08	0.05
DON	0.41	24.52	0.43	0.11

^a^*DM* dry matter

*Composition per kilogram: 250 g calcium, 30 g phosphorous, 85 g sodium, 35 g magnesium, 4000 mg zinc, 2000 mg manganese, 500 mg copper, 30 mg iodine, 10 mg cobalt, 20 mg selenium, 500,000 IU vitamin A, 80,000 IU vitamin D3, 500 mg vitamin E

BVDV antibody and antigen-negative bulls with an average initial body weight (b.w.) of 483 ± 46 kg were equipped with ear transponders for electronically recording of TMR and water intake, both offered for ad libitum consumption, via self-feeding stations (Insentec B.V., Marknesse, the Netherlands). Animals were kept in a thermally non-insulated and non-conditioned stable on slatted floor pens with 7–8 bulls per pen. Animals were randomly assigned to feeding and vaccination groups ensuring similar mean body weights.

Group CON comprised of 16 bulls. Eight bulls remained non-vaccinated and received physiological saline (mock-treated) while another eight animals of this feeding group were vaccinated to bovine viral diarrhea virus (BVDV) (*n* = 8) (Bovilis BVD-MD, MSD Animal Health, Schwabenheim an der Selz, Germany) into cervical muscles at day 1 and 21 (boost) of experiment. In feeding group FUS I, six bulls were mock-treated and eight bulls were BVDV-vaccinated; in groups FUS II and FUS III, the corresponding animal numbers amounted to eight and eight mock-treated and eight and seven BVDV-vaccinated.

Blood samples from a *Vena jugularis externa* were collected at days 0 (before first vaccination), 21 (before booster vaccination), and 28, 47, 56, and 68 of experiment. These samples were used for the present investigations.

### Sample preparation and analyses

#### Hematology and clinical chemistry

EDTA (ethylenediaminetetraacetic acid) blood was freshly used on the day of blood sampling. Heparinized plasma and serum samples were centrifuged at 1200×*g* for 15 min at 15 °C (Heraeus Varifuge® 3.0 R) and frozen (− 80 °C) until used for determination of clinical-chemical traits and antibodies.

An automatic hematology analyzer (Celltac-α, MEK 6450, Nihon Kohden Corporation, Tokyo, Japan) was used for determination of the red and white blood cell count in EDTA blood samples.

An automatic clinical chemistry analyzer (Eurolyser CCA180, Eurolab, Hallein, Austria) was used to determine various metabolites and enzyme activities in serum samples: non-esterified fatty acids (NEFA), beta-hydroxy butyrate (BHB), albumin, total protein, cholesterol, urea, aspartate aminotransferase (ASAT), glutamate dehydrogenase (GLDH), and gamma-glutamyltransferase (GGT).

#### Flow cytometry

The ability of the granulocytes and the PBMC to form reactive oxygen species (ROS) was examined using flow cytometry (FACS Canto II, BD Biosciences, San Jose, USA). The detection is based on the intracellular conversion of the non-fluorescent dye dihydrorhodamine 123 (DHR) to the fluorescent rhodamine 123 as described by Stelter et al. (2013). Tetradecanoyl-12,13-phorbol acetate (TPA) was used as a positive control because of its ability to trigger NADPH oxidase activity. Granulocytes and PBMCs were gated according to their size and granularity. At least 10,000 cells were recorded. Intracellular ROS formation is reported as percentage of ROS producing unstimulated (basal, granulocytes, and PBMC) or TPA-stimulated cells (granulocytes only) of total cells. Moreover, the ratio between the mean fluorescence intensity (MFI) of TPA-stimulated and unstimulated ROS-positive granulocytes was defined as stimulation index. Concentrations of unstimulated ROS-forming granulocytes and PBMC in blood were calculated as the product of their blood concentrations multiplied with the proportion of basal ROS producing cells.

Phenotypes of T cells were determined in EDTA samples. Cells were stained with monoclonal antibodies (mAbs) for CD4 (Mouse anti-bovine CD4: FITC), CD8 (Mouse anti-bovine CD8: RPE) or the corresponding isotype controls (Mouse IgG2a-negative control: RPE, Mouse IgG2b-negative control: FITC) at room temperature for 30 min. All antibodies were supplied by AbD serotec, Bio-Rad laboratories, Puchheim, Germany.

Erythrocytes were lysed using lysis buffer (BD FACS™ Lysing Solution, BD Biosciences, San Jose, USA) and then centrifuged in Hepes-buffered saline solution (HBS). For CD4+ and CD8+ T cell screening, the capture gate for the lymphocytes was set based on their side and forward scattering properties using a BD FACS Canto II. A total of at least 10,000 lymphocytes were counted and stored in list mode data files. Compensation of the spillover of both fluorochromes (FITC, PE) was performed by the BD FACS Diva software (BD Biosciences, San Jose, USA). The results are reported as a proportion of total lymphocytes, as ratio between CD4+ and CD8+ T cells and as absolute concentration in blood by multiplying the corresponding T cell proportions with total lymphocytes in blood.

#### Ferric reducing ability

FRA as a measure of the non-enzymatic anti-oxidative capacity of serum was analyzed according to Benzie and Strain (1996). Serum antioxidants convert Fe^3+^ tripyridyltriazine to Fe^2+^ tripyridyltriazine which is detected by the development of a blue color (kinetic measurement up to 15 min, extinction at 593 nm, 37 °C). FRA values are expressed as nanomole Fe^2+^ formed per milliliter of serum.

#### Antibodies against BVDV

Sera were taken at days 0 (before vaccination), 21, 28, 47, 56, and 70 and tested by a commercially available BVDV-antibody ELISA (ID Screen® BVD p80 Antibody Competition, ID.vet, France) according to the manufacturer’s instructions. Sera taken at the end of the study were additionally analyzed by a standard microneutralization test against BVDV-isolate NADL. Twofold dilutions of the sera (starting at 1/5) were prepared in Minimum Essential Medium (MEM). Fifty microliter of the diluted sera and 50 μl of MEM containing 100 tissue culture infectious dose 50% (TCID_50_) of BVDV were incubated in microtiter plates for 2 h. Thereafter, a KOP-R cell suspension (L244, Collection of Cell Lines in veterinary Medicine) in MEM containing 10% fetal calf serum was added and the plates were incubated for 72 h at 37 °C. Subsequently, the plates were fixed using heat treatment (2 h at 80 °C), incubated for 1 h at room temperature with a monoclonal anti-BVDV antibody (WB 103/105, AHVLA, UK), washed three times with PBS-Tween, and incubated for 1 h at room temperature with a peroxidase-labeled anti-mouse antibody (Sigma-Aldrich, Germany). The reaction was visualized by adding 3-Amino-9-ethylcarbazole (Sigma-Aldrich, Germany). All samples were tested in triplicate and the antibody titers were calculated as ND_50_ according to Behrens and Kaerber.

#### Statistics

Data excepting BVDV antibodies were analyzed using procedure MIXED implemented in the SAS software (Version 9.1, SAS-Institute-Inc., 2003, Cary, NC, USA) with feeding group (CON, FUS I, FUS II, FUSIII), time of blood sampling (0, 21, 28, 47, 56, and 68), vaccination (mock-treated, BVDV vaccinated), and all possible interactions as fixed factors. Zero samples were treated as co-variates to account for possible initial differences not accountable for treatment effects.

The effects of frequent measurements on the same bull were considered by a REPEATED statement. Treatment effects were considered as significant at probabilities (*p* values) ≤ 0.05 while a trend was acknowledged for probabilities between 0.05 and 0.1. Results are shown as least square means (LSMEANS) and pooled standard errors of means (PSEM) along with the *p* values for the fixed effects.

BVDV antibodies were expressed as the sample (S) optical density (OD) relative to negative (N) control OD (%) (S/N%). For statistical evaluation, these data were expressed as a proportion between 0 and 1 to enable parameter estimation by fitting the response distribution to the BETA-distribution. The LOGIT function served as link function. These modeling procedures are implemented in the GLIMMIX-procedure of SAS, which enables evaluation of generalized linear mixed models with random effects of none-Gaussian data with inhomogeneous variances.

Additionally, the relationships between all recorded variables were further examined by principal component analysis (PCA) based on correlations using the software package STATISTICA 12.0 (StatSoft, Inc. 2014, Tulsa, Oklahoma, USA). By using this method, it becomes possible to increase the interpretability of large datasets by reducing the dimensionality through creating new uncorrelated variables called principal components (PC) (Jolliffe and Cadima 2016). The dataset of the present experiment comprised a total of 42 variables which were subjected to correlation based extraction of PCs. As PCs successively extract variation from the dataset, the first two PCs are most informative for the whole dataset of the initial 42 variables.

## Results

### Toxin exposure

**Fig. 1:**
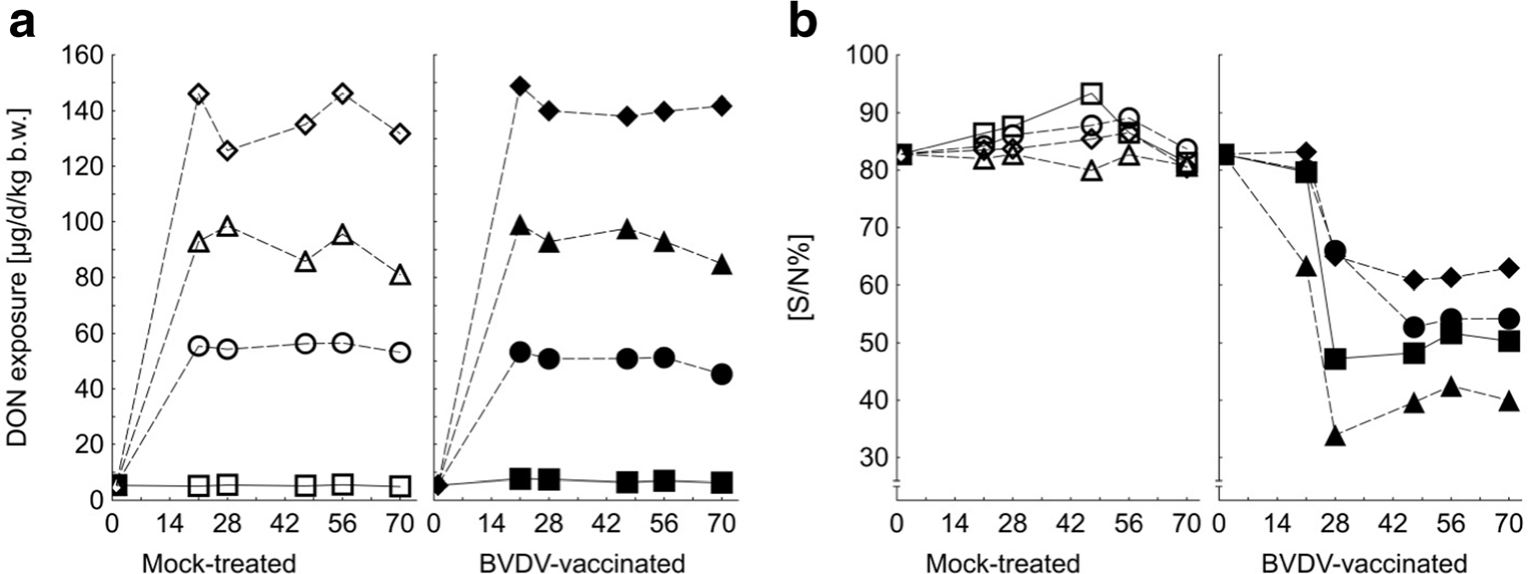
Time (experimental day)-dependent deoxynivalenol (DON) exposure (**a**) and antibody progression (S/N%: sample (S) optical density (OD) relative to negative (N) control OD (%)) in systemic blood of mock-treated bulls and bulls vaccinated against bovine viral diarrhea virus (BVDV) (**b**) depending on increasing dietary concentrations of *Fusarium* toxins. Mock-treated bulls were intramuscularly treated with saline and BVDV-vaccinated bulls with bovilis BVD-MD (MSD Animal Health, Schwabenheim an der Selz, Germany) at day 1 and 21 of experiment (least square means, *n* = 6–8). *P* values (DON exposure): group < 0.001, BVDV = 0.508, time < 0.001, group × BVDV = 0.208, time × group < 0.001, time × BVDV = 0.103, time × group × BVDV = 0.001; PSEM = 1.0 μg/kg b.w./d. *P* values (antibodies): group = 0.075, BVDV < 0.001, time = <0.001, group × BVDV = 0.108, time × group = 0.070, time × BVDV < 0.001, time × group × BVDV = 0.012; PSEM = 3.5%. *P* values were obtained from GLIMMIX-evaluated transformed data while symbols represent arithmetic mean values. The PSEM is based on a non-transformed scale

Toxin exposure reached a steady state after 3 weeks feeding the experimental diets and amounted on average to 6, 53, 92, and 139 μg DON per kilogram b.w. per day (Fig. 1a) and to 4, 6, 7, and 9 μg ZEN per kilogram b.w. per day (data not shown).

### Antibodies to BVDV

Sample (S) optical density (OD) relative to negative (N) control OD (%) (S/N%) remained unchanged in the course of the experiment in all mock-treated groups whereas a drop was noticed in all vaccinated groups which is indicative for antibody formation with efficiency being different for the feeding groups. No antibody formation was detected for groups CON, FUS I, and FUS III 2 weeks following first vaccination while group FUS II already displayed antibodies (Fig. 1b, Supplemental Table 1). One week after booster vaccination at week 3 of experiment, all groups had developed antibodies. The levels measured for the individual feeding groups remained constant for the last 6 weeks of the experiment albeit at different levels. Whilst group FUS II showed the strongest antibody response, group FUS III was characterized by the weakest antibody formation. Groups CON and FUS I ranged between the aforementioned feeding groups. The serum microneutralization test against BVDV isolate NADL which was performed at the end of the experiment as a confirmatory assay generally verified the ELISA results. Thus, all samples which were tested positive in the ELISA assay were also confirmed to be positive in the serum microneutralization test.

### Hematology

#### White blood count

**Fig. 2:**
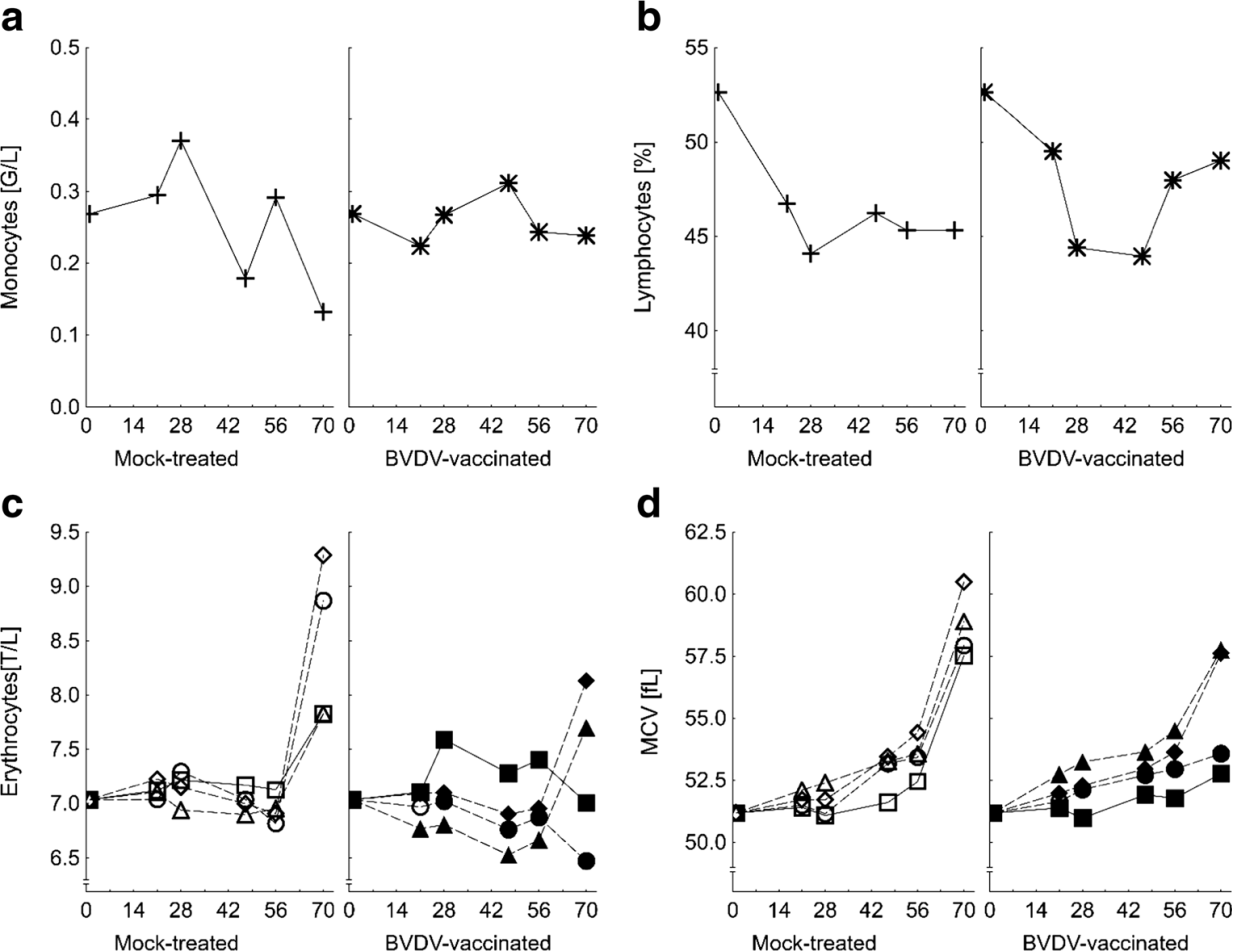
Monocyte counts (**a**), lymphocyte proportions (**b**), erythrocyte counts (**c**), and mean corpuscular volume (MCV) (**d**) of mock-treated bulls and bulls vaccinated against bovine viral diarrhea virus (BVDV) depending on increasing dietary concentrations of *Fusarium* toxins and on time (experimental day). Mock-treated bulls were intramuscularly treated with saline and BVDV-vaccinated bulls with bovilis BVD-MD (MSD Animal Health, Schwabenheim an der Selz, Germany) at day 1 and 21 of experiment (least square means, *n* = 6–8, *p* values, and PSEM in Table 2 and Table 3)

Counts of total leucocytes, lymphocytes, and eosinophil granulocytes decreased significantly in the course of the experiment while those of neutrophil granulocytes increased at the same time (Table 2). Monocyte counts kept stable over time in vaccinated groups while a decrease was noticed in mock-treated groups irrespective of feeding group (*p*_time × BVDV_ = 0.015) (Fig. 2a). Lymphocyte proportions decreased during the first 3 weeks of the experiment in mock-treated groups and kept stable thereafter for the rest of the experiment while an initial decrease was followed by a final increase up to the initial levels in all vaccinated groups (*p*_time × BVDV_ = 0.034) (Fig. 2b).

**Table 2:** White blood count of bulls fed diets with increasing concentrations of *Fusarium* toxins (Deoxynivalenol, DON; zearalenone, ZEN); half of each feeding group remained either non-vaccinated or was vaccinated to bovine viral diarrhea virus (BVDV) (least square means, *n* = 6–8)

Group	DON/ZEN(mg/kg DM)	BVDV vaccination^a^	Time [experimental day]	Leucocytes [G/l]	Lymphocytes [G/L^b^]	Monocytes [G/L]	Neutrophil granulocytes [G/L]	Eosinophil granulocytes [G/L]	Lymphocytes [%]	Neutrophil granulocytes [%]
			0	8.04	4.20	0.28	2.69	0.87	52.3	33.2
CON	0.36/0.08	–	70	7.66	3.39	0.10	3.46	0.71	46.0	42.7
FUS I	3.01/0.28	–	70	6.60	2.76	0.18	3.22	0.44	42.5	47.4
FUS II	5.66/0.48	–	70	6.58	3.31	0.16	2.34	0.76	49.9	35.6
FUS III	8.31/0.69	–	70	7.88	3.39	0.16	3.88	0.45	43.4	49.2
CON	0.36/0.08	+	70	6.37	3.07	0.15	2.82	0.33	48.3	42.9
FUS I	3.01/0.28	+	70	7.00	3.50	0.18	2.71	0.61	51.9	35.9
FUS II	5.66/0.48	+	70	8.19	4.17	0.30	2.84	0.88	51.5	35.1
FUS III	8.31/0.69	+	70	7.46	3.34	0.24	3.27	0.60	44.8	43.8
*P* values										
Group				0.124	0.318	0.459	0.329	0.163	0.316	0.208
BVDV				0.837	0.723	0.917	0.362	0.863	0.245	0.269
Time				0.001	< 0.001	< 0.001	< 0.001	< 0.001	< 0.001	< 0.001
Group × BVDV				0.543	0.226	0.957	0.759	0.062	0.711	0.604
Time × group				0.154	0.817	0.825	0.249	0.669	0.718	0.450
Time × BVDV				0.520	0.266	0.015	0.306	0.861	0.034	0.447
Time × group x BVDV				0.385	0.271	0.932	0.377	0.486	0.361	0.684
PSEM^c^				0.2	0.1	0.0	0.2	0.1	1.2	1.7

^a^“-“Bulls were not vaccinated; “+” bulls were vaccinated against BVDV (Bovilis BVD-MD, MSD Animal Health, Schwabenheim an der Selz, Germany) into cervical musculature at day 1 and 21 (boost) of experiment; ^b^G/L, Giga/Liter = 10^9^ cells/L; ^c^PSEM, pooled standard error of means

#### Red blood count

**Fig. 3:**
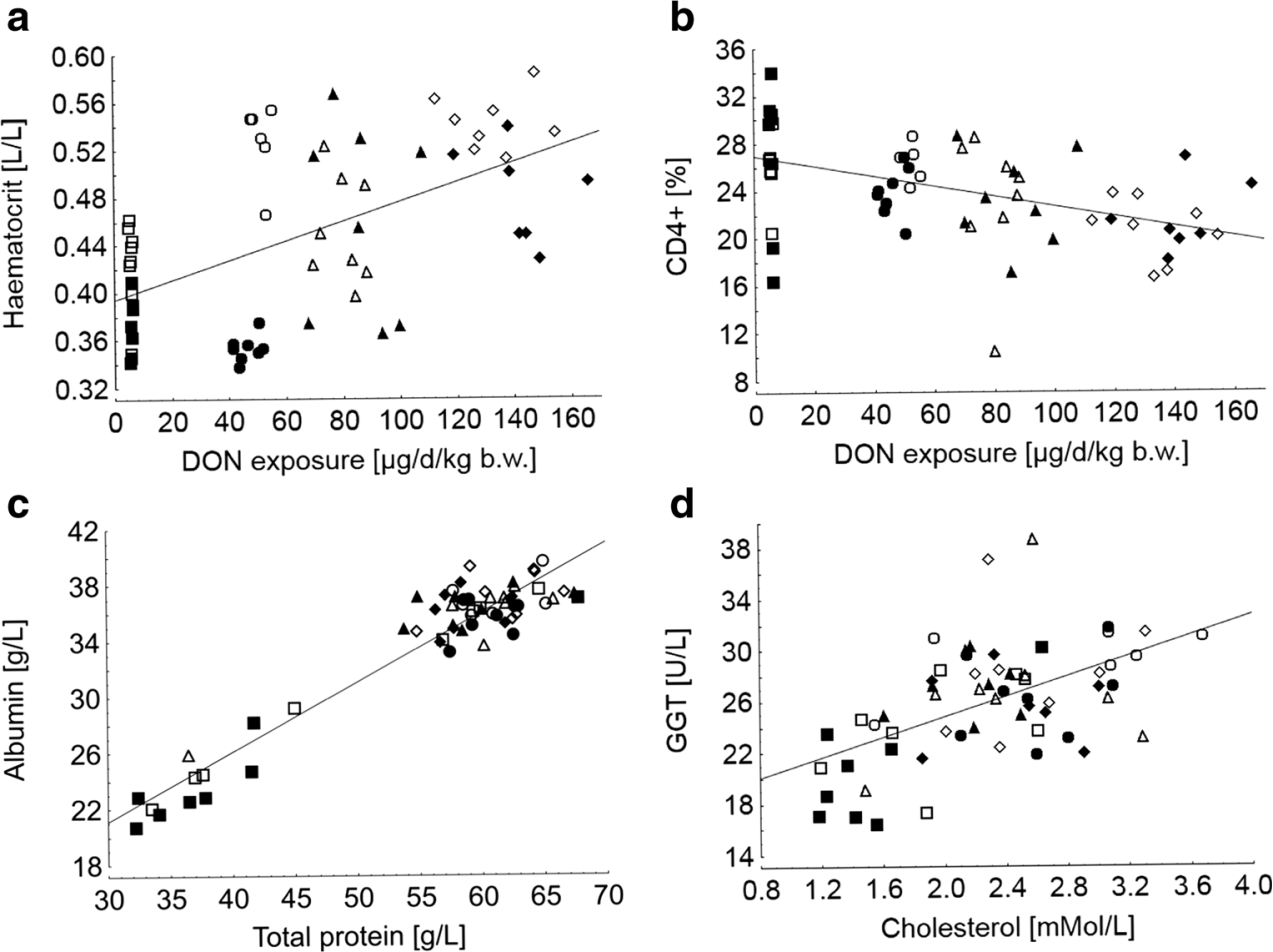
Hematocrit (**a**), and proportion of CD4+ cells in peripheral blood (**b**) depending on deoxynivalenol (DON) exposure, relationships between albumin and protein concentration (**c**), and gamma-glutamyltransferase (GGT) activity and cholesterol concentration in blood (**d**) at the end of the experiment (week 10 of experiment). Hematocrit = 0.394 + 0.0008·DON exposure; *r*^2^ = 0.297, RSD = 0.06 L/L (*p* < 0.05, *N* = 60) CD4**+** = 26.9–0.042·DON exposure; *r*^2^ = 0.239, RSD = 3.8% (*p* < 0.05, *N* = 60) Albumin = 6.2 + 0.5·protein; *r*^2^ = 0.904, RSD = 1.6 g/L (*p* < 0.05, *N* = 61) GGT = 16.9 + 3.95·cholesterol; *r*^2^ = 0.288, RSD = 3.1 U/L (*p* < 0.05, *N* = 61)

Red blood cell count was markedly influenced by all fixed factors (feeding group, vaccination, and time) in an interacting manner excepting the red cell distribution width which remained unaltered (Table 3). Erythrocyte counts remained stable over time until week 8 of the experiment and increased thereafter whereby this increase was more pronounced in mock-treated groups than in vaccinated groups and additionally steeper in groups fed the FUS-contaminated diets; particularly in group FUS III (*p*_time × group × BVDV_ < 0.001) (Fig. 2c). Similar significant relationships were found for hematocrit. In addition, a significant positive linear association was found between DON exposure and hematocrit (Fig. 3a). Hemoglobin concentration decreased time-dependently in the mock-treated CON group while an opposite trend was observed for the vaccinated counterpart. Moreover, opposite relationships were observed for group FUS I, i.e., a time-dependent increase in mock-treated and a corresponding decrease in vaccinated bulls. All these interactive relationships gave rise to the significant interactions between time, feeding group, and vaccination (*p*_time × group × BVDV_ < 0.001). Mean corpuscular volume (MCV) increased over time but with feeding group and vaccination-dependent efficiencies (*p*_time × group_ < 0.001, (*p*_time × BVDV_ < 0.001). The increase was generally more pronounced in mock-treated groups whereby the increase was less steep in CON group compared to the FUS groups (Fig. 2d). Mean corpuscular hemoglobin (MCH) remained stable until week 8 of experiment and decreased thereafter in all mock-treated feeding groups whilst it remained constant in groups CON and FUS I (*p*_time × group × BVDV_ < 0.001).

**Table 3:** Red blood count of bulls fed diets with increasing concentrations of *Fusarium* toxins (Deoxynivalenol, DON; zearalenone, ZEN); half of each feeding group remained either non-vaccinated or was vaccinated to bovine viral diarrhea virus (BVDV) (least square means, *n* = 6–8)

Group	DON/ZEN[mg/kg DM]	BVDV vaccination^a^	Time [experimental day]	Erythrocytes [T/L^b^]	Hemoglobin [mMol/L^c^]	Hematocrit [L/L]	Mean corpuscular volume [MCV, fL^d^]	Mean corpuscular hemoglobin [MCH, fMol^e^]	Red cell distribution width [%]
			0	7.0	7.0	0.36	51.3	1.00	14.2
CON	0.36/0.08	–	70	7.6	6.4	0.43	56.1	0.85	14.8
FUS I	3.01/0.28	–	70	8.7	7.1	0.52	60.5	0.82	14.1
FUS II	5.66/0.48	–	70	7.8	6.7	0.45	58.4	0.87	14.7
FUS III	8.31/0.69	–	70	8.8	7.0	0.54	61.8	0.80	14.7
CON	0.36/0.08	+	70	7.3	7.2	0.38	52.0	0.99	14.7
FUS I	3.01/0.28	+	70	6.5	6.7	0.35	54.4	1.03	14.5
FUS II	5.66/0.48	+	70	8.1	6.9	0.46	56.5	0.86	14.5
FUS III	8.31/0.69	+	70	8.3	7.1	0.48	58.3	0.86	14.7
*P* values									
Group				0.028	0.171	0.003	< 0.001	< 0.001	0.393
BVDV				0.003	0.197	0.001	0.096	0.950	0.954
Time				<0.001	< 0.001	< 0.001	0.249	0.099	0.189
Group × BVDV				0.150	0.001	0.009	0.433	0.114	0.588
Time × group				< 0.001	< 0.001	< 0.001	< 0.001	< 0.001	0.901
Time × BVDV				< 0.001	0.100	< 0.001	< 0.001	< 0.001	0.917
Time × group × BVDV				< 0.001	< 0.001	< 0.001	0.340	< 0.001	0.368
PSEM^f^				0.1	0.1	0.004	0.4	0.01	0.1

^a^“-“bulls were not vaccinated; “+” bulls were vaccinated against BVDV (Bovilis BVD-MD, MSD Animal Health, Schwabenheim an der Selz, Germany) into cervical musculature at day 1 and 21 (boost) of experiment; ^b^T/L, Tera/Liter = 10^12^ cells/L; ^c^mMol/L, millimol/Liter; ^d^fL, femtolitre; ^e^fMol, femtomol; ^f^PSEM, pooled standard error of means

### Flow cytometry

#### T cell phenotyping

**Fig. 4:**
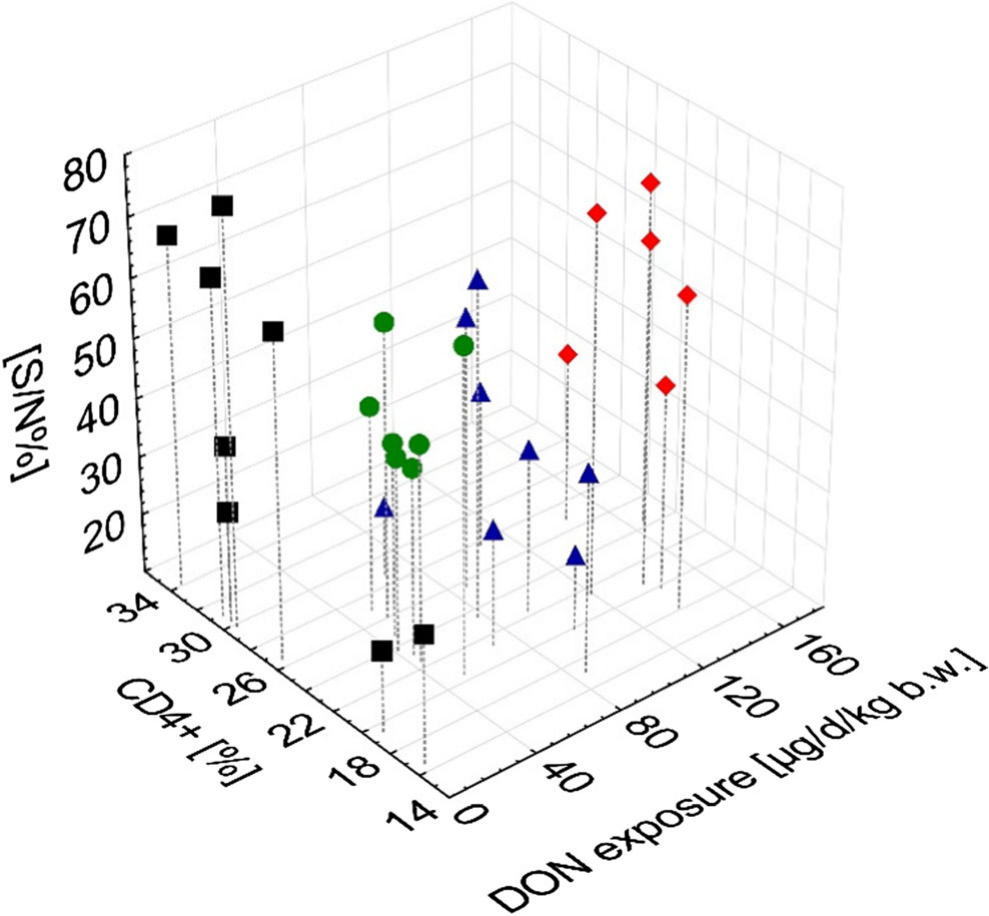
Relationships between deoxynivalenol (DON) exposure, proportions of CD4+ T cells and antibody titers to bovine viral diarrhea virus (BVDV) (S/N%) at day 70 of experiment in BVDV-vaccinated bulls (Bovilis BVD-MD (MSD Animal Health, Schwabenheim an der Selz, Germany, day 1 and 21 of experiment)

T cell phenotyping revealed no time-dependent change in the CD4+ proportion in group FUS III while an increase was noticed for the CON group (*p*_time × group_ < 0.007) (Table 4). Moreover, a significant negative linear relationship between DON exposure and CD4+ proportions was identified at week 10 of experiment (Fig. 3b). Particularly low DON exposures (Groups FUS I and FUS II) not only decreased the proportion of CD4+ T cells but also increased the antibody titers to BVDV while the highest DON exposure (Group FUS III) was accompanied by a marked drop in antibody titers (Fig. 4).

**Table 4:** Relative proportions of CD4+ and CD8+ cells of total lymphocytes and total counts in peripheral systemic blood of bulls fed diets with increasing concentrations of *Fusarium* toxins (Deoxynivalenol, DON; zearalenone, ZEN); half of each feeding group remained either non-vaccinated or was vaccinated to bovine viral diarrhea virus (BVDV) (least square means, *n* = 6–8)

Group	DON/ZEN (mg/kg DM)	BVDV vaccination^a^	Time (experimental day)	CD4+ (%)	CD8+ (%)	CD4+/CD8+	CD4+ (G/L^b^)	CD8+ (G/L)
			0	21.9	14.6	1.6	0.91	0.60
CON	0.36/0.08	–	70	26.6	14.9	1.9	0.89	0.50
FUS I	3.01/0.28	–	70	26.5	19.5	1.4	0.73	0.53
FUS II	5.66/0.48	–	70	23.0	11.2	2.1	0.74	0.37
FUS III	8.31/0.69	–	70	20.7	13.9	1.7	0.70	0.47
CON	0.36/0.08	+	70	26.2	14.6	1.9	0.77	0.44
FUS I	3.01/0.28	+	70	23.9	15.7	1.6	0.84	0.54
FUS II	5.66/0.48	+	70	23.3	15.2	1.6	0.96	0.65
FUS III	8.31/0.69	+	70	21.6	12.1	1.8	0.73	0.42
*P* values								
Group					0.022	0.208	0.398	0.820
BVDV					0.635	0.898	0.302	0.330
Time				< 0.001	< 0.001	< 0.001	< 0.001	< 0.001
Group × BVDV				0.833	0.065	0.211	0.026	0.027
Time × group				0.007	0.022	0.208	0.398	0.820
Time × BVDV				0.984	0.635	0.898	0.302	0.330
Time × group × BVDV				0.833	0.065	0.211	0.026	0.027
PSEM^c^				0.7	0.7	0.1	0.04	0.03

^a^“-“bulls were not vaccinated; “+” bulls were vaccinated against BVDV (Bovilis BVD-MD, MSD Animal Health, Schwabenheim an der Selz, Germany) into cervical musculature at day 1 and 21 (boost) of experiment; ^b^G/L, Giga/Liter = 10^9^ cells/L; ^c^PSEM, pooled standard error of means

For the proportion of CD8+ T cells, an increase was observed (*p*_time × group_ = 0.022) although it appeared that the feeding group differences were smaller in the vaccinated groups compared to the mock-treated counterparts (*p*_time × group × BVDV_ = 0.065). The CD4+ to CD8+ ratio significantly increased over time (*p* < 0.001) but was not influenced by other factors. Total CD4+ counts decreased in mock-treated FUS I group whilst it remained constant in the corresponding vaccinated group. Opposite relationships were noticed in the CON group (*p*_time × group × BVDV_ = 0.026). The time-dependent decrease in total CD8+ counts was more pronounced in mock-treated FUS II group compared to their vaccinated counterparts. For the CON group, opposite effects were observed (*p*_time × group × BVDV_ = 0.027).

#### Functionality of PMN and PBMC

The proportion of granulocytes forming ROS basally first increased and later decreased to the initial levels while the corresponding total counts decreased over time (Table 5). The percentage of granulocytes mounting an oxidative burst after TPA stimulation increased in the course of the experiment as the stimulation index did. The basal ROS forming activity of PBMC and the corresponding total counts did not change markedly over time and were not influenced by feeding group and vaccination status, except for the markedly higher values measured in mock-treated group FUS II at week 7, and in the vaccinated group FUS I at week 10 of experiment (*p*_time × group × BVDV_ < 0.001).

**Table 5:** Reactive oxygen species (ROS) formation of peripheral blood mononuclear cells (PBMC) and of total granulocytes (GR) and ability of GR to respond to a stimulus ex vivo. Blood samples were collected from bulls fed diets with increasing concentrations of *Fusarium* toxins (Deoxynivalenol, DON; zearalenone, ZEN); half of each feeding group remained either non-vaccinated or was vaccinated to bovine viral diarrhea virus (BVDV) (least square means, *n* = 6–8)

Group	DON/ZEN (mg/kg DM)	BVDV vaccination^a^	Time (experimental day)	ROS formation					GR stimulation index^b^
				Basal (unstimulated)				TPA-stimulated	
				ROS+ GR (%)^c^	ROS+ GR (G/L)^c^	ROS+ PBMC (%)^d^	ROS + PBMC (G/L)^d^	ROS+ GR (%)	
			0	6.4	0.22	2.94	0.14	96.9	22.5
CON	0.36/0.08	–	70	7.8	0.38	0.83	0.03	99.6	23.1
FUS I	3.01/0.28	–	70	4.6	0.16	1.57	0.05	99.6	24.6
FUS II	5.66/0.48	–	70	5.9	0.19	3.18	0.10	98.6	23.3
FUS III	8.31/0.69	–	70	4.1	0.18	1.84	0.06	99.1	29.1
CON	0.36/0.08	+	70	6.5	0.19	2.83	0.09	99.3	30.3
FUS I	3.01/0.28	+	70	9.9	0.30	6.46	0.23	99.4	11.0
FUS II	5.66/0.48	+	70	6.3	0.22	1.76	0.06	99.3	31.0
FUS III	8.31/0.69	+	70	4.0	0.16	1.45	0.05	98.6	28.9
*P* values									
Group				0.141	0.531	0.001	< 0.001	0.277	0.752
BVDV				0.260	0.710	0.445	0.291	0.995	0.582
Time				< 0.001	0.002	< 0.001	< 0.001	< 0.001	0.013
Group × BVDV				0.360	0.337	< 0.001	< 0.001	0.438	0.515
Time × group				0.344	0.775	< 0.001	< 0.001	0.226	0.625
Time × BVDV				0.512	0.920	0.008	0.008	0.981	0.910
Time × group × BVDV				0.592	0.573	< 0.001	< 0.001	0.472	0.590
PSEM^e^				0.6	0.03	0.5	0.01	0.1	3.3

^a^“-“bulls were not vaccinated; “+” bulls were vaccinated against BVDV (Bovilis BVD-MD, MSD Animal Health, Schwabenheim an der Selz, Germany) into cervical musculature at day 1 and 21 (boost) of experiment; ^b^Ratio between TPA (tetradecanoyl-12,13-phorbol acetate) -stimulated and unstimulated ROS+ GR; ^c^ Peripheral blood granulocytes exhibiting a basal ROS production, G/L, Giga/Liter = 10^9^ cells/L; ^d^Peripheral blood mononuclear cells exhibiting a basal ROS production; ^e^PSEM, pooled standard error of means

#### Serum clinical-chemical traits

Serum BHB levels increased markedly in vaccinated groups FUS II and FUS III at week 10 of experiment while a decrease was noticed for CON group (Table 6). These changes were less variable in mock-treated groups (*p*_time × group_ < 0.001, *p*_group × BVDV_ = 0.019). NEFA serum levels decreased over time irrespective of vaccination and feeding group. Cholesterol concentrations in serum increased in the course of the experiment in mock-treated FUS III group while a drop was noticed for vaccinated and mock-treated CON group (*p*_time × group × BVDV_ < 0.001). Albumin concentration in serum exclusively decreased in both CON groups while no longitudinal change occurred in all other feeding groups (*p*_time × group × BVDV_ < 0.001). Similar relationships were found for the total serum protein content. Moreover, about 90% of the variation in albumin concentration could be explained by total protein concentration (Fig. 3c). The increase of the corresponding linear regression suggests that the albumin proportion of total protein amounts to 50% on average independent of time and treatments. After 7 weeks, a maximum of serum urea concentration was found whose magnitude was more pronounced in all three FUS groups, particularly in mock-treated groups. Glutamate-dehydrogenase (GLDH) activity in serum increased time-dependently both in mock-treated and vaccinated FUS III group while a decrease was detected for both CON groups (*p*_time × group_ < 0.001). Gamma-glutamyl transferase (GGT) activity of mock-treated FUS III group increased with time while the activity decreased in the vaccinated CON group. The activities of the remaining groups did not show time-dependencies (*p*_time × group_ = 0.003, *p*_time × BVDV_ = 0.005). GGT increased linearly with cholesterol concentration in serum (Fig. 3c). Aspartate-aminotransferase (ASAT) activity decreased with time in both CON groups with a similar trend for both FUS I and FUS II groups while this enzyme activity was kept stable in mock-treated and vaccinated FUS III group (*p*_time × group_ < 0.001). Ferric reducing ability (FRA) of serum was characterized by a decrease in week 7, and an increase until the end of the experiment where initial levels were reached. BVDV vaccination tended to increase serum FRA, particularly at day 70 of experiment and to increase the time-dependent variability amongst treatment groups (*p*_time × group × BVDV_ = 0.060).

**Table 6:** Clinical-chemical characteristics of bulls fed diets with increasing concentrations of *Fusarium* toxins (Deoxynivalenol, DON; zearalenone, ZEN) half of each feeding group remained either non-vaccinated or was vaccinated to bovine viral diarrhea virus (BVDV) (least square means, *n* = 6–8)

Group	DON/ZEN (mg/kg DM)	BVDV vaccination^a^	Time (experimental day)	BHB (mMol/L)	NEFA (mMol/L)	Cholesterol (mMol/L)	Albumin (g/L)	Total protein (g/L)	Urea (mMol/L)	ASAT (U/L)	GGT (U/L)	GLDH (U/L)	Fe (II) (μM)^b^
			0	0.348	0.275	2.2	36.0	59.5	5.1	58.4	24.7	8.8	238
CON	0.36/0.08	–	70	0.304	0.239	2.0	30.5	49.2	5.0	44.5	24.4	7.1	222
FUS I	3.01/0.28	–	70	0.401	0.241	2.7	36.9	61.8	6.9	53.7	28.9	11.1	232
FUS II	5.66/0.48	–	70	0.418	0.200	2.4	35.2	58.4	5.4	55.2	26.9	9.0	237
FUS III	8.31/0.69	–	70	0.268	0.265	2.5	37.2	61.9	6.2	61.3	28.1	11.8	228
CON	0.36/0.08	+	70	0.226	0.238	1.6	26.2	43.2	4.0	42.1	21.4	6.7	237
FUS I	3.01/0.28	+	70	0.345	0.215	2.6	35.6	60.5	5.1	50.1	26.2	8.2	254
FUS II	5.66/0.48	+	70	0.532	0.198	2.2	36.3	59.1	5.5	47.6	27.1	7.9	228
FUS III	8.31/0.69	+	70	0.474	0.191	2.5	36.1	58.7	5.9	56.0	25.5	12.0	239
*P* values													
Group				0.038	0.423	< 0.001	0.002	0.001	0.008	0.003	0.009	< 0.001	0.310
BVDV				0.698	0.863	0.010	0.880	0.633	0.014	0.034	0.069	0.032	0.083
Time				< 0.001	< 0.001	0.170	< 0.001	< 0.001	< 0.001	< 0.001	< 0.001	< 0.001	< 0.001
Group × BVDV				0.019	0.423	0.019	0.486	0.474	0.444	0.924	0.185	0.762	0.067
Time x group				< 0.001	0.554	< 0.001	< 0.001	< 0.001	0.045	< 0.001	0.003	< 0.001	0.806
Time × BVDV				0.158	0.172	0.008	0.071	0.407	0.109	0.071	0.005	0.150	0.146
Time × group × BVDV				0.107	0.935	0.027	0.029	0.241	0.647	0.614	0.329	0.591	0.060
PSEM^c^				0.02	0.017	0.1	0.6	1.1	0.5	1.5	0.7	0.5	4

^a^“-“bulls were not vaccinated; “+” bulls were vaccinated against BVDV (Bovilis BVD-MD, MSD Animal Health, Schwabenheim an der Selz, Germany) into cervical musculature at day 1 and 21 (boost) of experiment. ^b^ Ferric reducing ability (FRA) of serum; ^c^ PSEM, pooled standard error of means

Further abbreviations: BHB, 3-β-hydroxybutyrate; NEFA, non-esterified fatty acids; GGT, gamma-glutamyl transferase, U/L = units/l; ASAT, aspartate-aminotransferase; GLDH, glutamate-dehydrogenase.

#### Principal component analysis

**Fig. 5:**
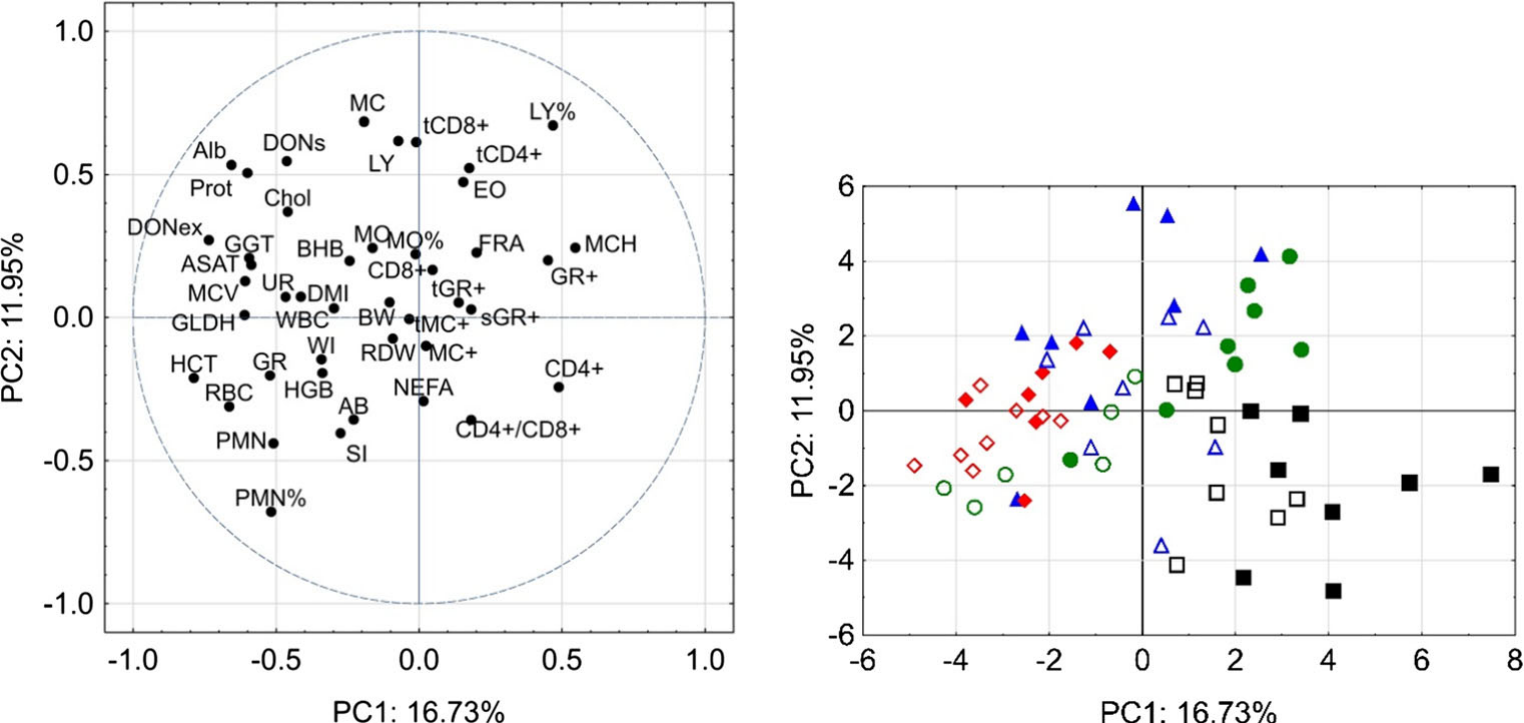
Principal component analysis for a two-dimensional visualization of the relationships between 42 variables collected from the experiment at day 68: variables for the analysis: *WBC* white blood cell count, *LY%* lymphocyte proportion, *LY* lymphocyte concentration, *MO%* monocyte proportion, *MO* monocyte concentration, *MC* concentration of mononuclear cells, *PMN%* neutrophil granulocyte proportion, *PMN* neutrophil granulocyte concentration, EO eosinophil granulocyte concentration, *GR* total granulocyte concentration, *RBC* red blood cell count, *HGB* hemoglobin, *HCT* hematocrit, *MCV* mean corpuscular volume, *MCH* mean corpuscular hemoglobin, *RDW* red cell distribution width, *Alb* albumin, *Chol* cholesterol, *ASAT* aspartate-aminotransferase, *GGT* gamma-glutamyl transferase, *GLDH* glutamate-dehydrogenase, *Prot* protein, *UR* urea, *BHB* 3-β-hydroxybutyrate, *NEFA* non-esterified fatty acids, *CD4+* CD4+ cells of total lymphocytes; *tCD4+* total concentration of CD4+ cells, *CD8+* CD8+ cells of total lymphocytes, *tCD8+* total concentration of CD8+ cells, *CD4+/CD8+* ratio between CD4+ and CD8+ cells, *GR+* proportion of reactive oxygen species (ROS) positive granulocytes, *tGR+* concentration of ROS positive granulocytes, *sGR+* proportion of TPA (tetradecanoyl-12,13-phorbol acetate)-stimulated ROS+ GR, *SI* ratio between TPA-stimulated and unstimulated ROS+ GR, *MC+* proportion of ROS positive MC, *tMC+* concentration of ROS positive MC, *DONex* daily deoxynivalenol (DON) exposure, *DONs* DON and de-epoxy-DON concentration in blood, *DMI* dry matter intake, *WI* water intake, *BW* body weight, *AB* antibodies against bovine viral diarrhea virus (BVDV). Left—projection of variables, right—projection of cases (individual bulls)

Correlation-based PCA was employed to further investigate the relationships between 42 parameters recorded for each animal in the experiment. The results showed that the first two components (PC 1 and PC 2) extracted approximately 29% of the total variance (Fig. 5). The visualized relationship between the step-by-step extracted components and the corresponding eigenvalues (scree plot) did not indicate a distinct break point which separates the more from the less important components. Rather, the mean value of all 42 eigenvalues of 1.0 corresponded to a total of 13 extracted components which explained approximately 83% of the total variance.

At first, all 42 variables were plotted in the space spanned between PC1 and PC2 in order to examine their relationships to each other and to both components. As the PCA was based on correlations, a localization of a particular variable in the center formed by the cross between the zero correlations of PC1 and PC2 means a poor or no correlation of that variable to both components. On the other hand, localizing at the circle indicates a correlation to either PC1 or PC2 of 1.0 or -1.0 when the counterpart PC equals zero.

Albumin and total protein concentrations in blood are located close to each other confirming the strong linear relationship between these two variables (see also Fig. 3c). Moreover, DON exposure was clearly associated with the resulting DON concentration in serum and formed a cluster with albumin, total protein, cholesterol, and the liver lesion indicating enzymes GGT, GLDH, and ASAT.

Moreover, the negative correlation between DON exposure and CD4+ T cells is also mirrored in the PCA plot as they were opposed correlated to PC 1 and thus distantly located in the plot. Red blood count indicators formed another cluster together with functional parameters of PMN and the BVDV-antibody titers.

Projecting the cases into the component space is another feature of the PCA and allows to identify individuals which are more similar than others. Here, all animals of both CON groups formed a distinguishable cluster while the FUS groups obviously belonged to another cluster. A closer look to the latter cluster revealed that these FUS groups also formed sub-clusters although the transition between them was more fluent. Additionally, the mock-treated groups were distinguishable from their vaccinated counterparts within the same feeding group whereby this effect was more obvious for group FUS I than for the other groups.

## Discussion

BVDV vaccines usually contain either modified-live virus (MLV) or inactivated virus. While MLV stimulates both cell-mediated and humoral immunity-inactivated vaccine results in a weaker T cell response but a strong humoral immunity as mediated by adjuvants (Stevens et al. 2009). Vaccination with MLV is known to induce transient immunosuppression due to replication of the MLF in immune cells (Ridpath 2013). As we used an inactivated vaccine, such effects were not expected. However, vaccination interactively influenced several leucocyte parameters. The time-constancy of monocyte counts in BVDV-vaccinated bulls compared to the final decline in their mock-treated counterparts might be interpreted as a general stimulation of the immune system probably mediated by the adjuvant and further triggered by the interactions between vaccine antigen and antigen-presenting cells. Similarly, the higher lymphocyte proportions in vaccinated bulls, particularly after initial and booster vaccination, might be a reflection of a generally stimulated proliferative response. In interpreting the effects of vaccination on the immune system, it needs to be considered that the readouts represented both an antigen-specific response, i.e., the BVDV-antibody titers, but also unspecific immune reactions. These rather antigen-unspecific effects might result from the adjuvant components of the BVDV vaccine. According to the declaration of the manufacturer, the vaccine preparation used in the present experiment contained aluminum (III)-ion (Al^3+^) as aluminum phosphate and aluminum hydroxide as adjuvant and methyl-para-hydroxy-benzoate as preservation agent. One vaccination dose of the used vaccine contained 6–9 mg Al^3+^ and corresponded to a single dose of approximately 12–19 μg of Al^3+^ per kilogram b.w. at day 0 and 21 of experiment.

While the proportion of CD4+ T cells increased in the vaccinated control group over time, the percentage of this phenotype remained constant in the group exposed to the highest levels of *Fusarium* toxins (FUS III). Opposite relationships were observed for cytotoxic T cells (CD8+) with a decrease in FUS III group and a time-constancy in the control group resulting both in a comparable CD4+ to CD8+ ratio and total counts of both phenotypes in peripheral blood. As aluminum adjuvants trigger humoral immunity by activating Th2 type cells (Gupta 1998) the proportional increase in CD4+ T cells of the control group might be due to an increase in Th2 type cells supporting antibody response by B cells. In addition, recent research on hepatitis B vaccines suggests that aluminum-based adjuvants improve both Th1 and Th2 cellular responses to antigens (He et al. 2015). Therefore, the discussed increase in CD4+ cells as observed in the present experiment could be due to increases both of Th1- and Th2-type cells.

Obviously, such an effect was prevented by high exposure to *Fusarium* toxins resulting in a lower BVDV-antibody response in group FUS III compared to the control group. However, the dose-response relationships between *Fusarium* toxin or DON exposure and BVDV-antibody formation appeared to be non-linear since the medium exposure level (FUS II) induced the most strongest BVDV-antibody response which additionally was paralleled by mean CD4+ T cell proportions lower than in the control group but higher than in group FUS III. Although the relationship between DON exposure and the proportion of CD4+ T cells appeared to be linear (Fig. 3), the BVDV-antibody response was non-linear (Figs. 1 and 4) suggesting additional mechanisms other than the involvement of CD4+ T cells. In interpreting the relationships between DON-exposure and BVDV-antibody formation, it has to be considered that we used a competitive ELISA which detects all antibody isotypes. Therefore, the net outcome of total antibody response might be influenced by DON exposure mediated linear and non-linear responses of specific isotypes.

It is interesting to note that a decrease in CD4+ T cells was also reported for early lactating dairy cows exposed to increasing dietary DON concentrations up to 5.2 mg/kg DM (Dänicke et al. 2017). These and present results are anyway surprising since ingested DON is known to be largely metabolized to de-DON in the rumen resulting in serum de-DON levels substantially higher than that of non-metabolized DON (Dänicke and Brezina 2013). Also, for the present experiment, nearly exclusively de-DON was detected in blood of bulls in a linear dose-response-related fashion. The ratio between DON and de-DON amounted to approximately 1 to 40 corresponding to a de-DON proportion of 95% of the sum of DON plus de-DON in plasma (Winkler et al. 2016). With regard to comparative in vitro toxicity using bovine PBMC, an IC_50_ (i.e., the concentration where the proliferation was inhibited by 50%) of 0.5 μM was titrated for DON while the maximum tested de-DON concentration of 18.29 μM inhibited proliferation just by 24% underpinning the low toxicity of this rumen-originating DON metabolite on this particular immune cell population. For cows exposed to a *Fusarium*-toxin contaminated diet for several weeks maximum serum de-DON levels of 52 ng/mL (0.19 μM) were detected while DON concentrations reached maximum levels of 9 ng/mL (0.03 μM) (Dänicke et al. 2011). PBMC isolated from these cows were characterized by a slightly lower viability ex vivo compared to PBMC of non-exposed control cows although blood toxin levels were markedly lower than the effective doses found in the in vitro trial. Besides the presence of *Fusarium* toxins other than DON and ZEN in the contaminated diet, further associated observations were discussed as possible reasons for the unexpected effects. Intake of DM and consequently of energy, nutrients, and mycotoxins was increased in cows fed the contaminated diet (Keese et al. 2008a) and suspected to have influenced the vulnerability of PBMC (Dänicke et al. 2011). Bulls of groups FUS I, II, and III consumed approximately 2–4%, 3–6%, and 8–10%, respectively, more DM, metabolizable energy and crude protein compared to animals of the CON group (Winkler et al. 2016) supporting the view that an increased metabolic supply with nutrients, energy but also with mycotoxins interferes with immunological responses. The linear relationships between blood cholesterol and GGT activity, total protein, and albumin, but also between DON-exposure and hematocrit (Fig. 3) as well as the higher blood urea concentration in all FUS groups further support the view that a higher DM intake influences metabolic processes and presumably has consequences for the immune system. This aspect becomes particularly obvious in group FUS III where the increase in energy and nutrient intake was most pronounced. Metabolic parameters further support this view as this group exhibited a high-blood level of BHB compared to the CON group which might indicate a higher rumen turnover due to higher feed intake which results in higher levels of ruminal butyrate subsequently metabolized to BHB. Moreover, NEFA levels were lower in group FUS III suggesting a lower level of lipolysis compared to CON group. However, these changes were particularly observed in BVDV-vaccinated groups whereas the time-dependent alterations in mock-treated groups were rather subtle. In addition, for group FUS III, the highest activities of GGT, GLDH, and ASAT in blood were detected leading to conclude a higher degree of liver lesions caused by the highest exposure to DON and/or by the stimulated DM intake compared to the other groups. However, it needs to be stressed that enzyme activities of all treatment groups were generally lower than the reference limits of 50, 30, and 80 U/L for GGT, GLDH, and ASAT (Moritz 2014), respectively. Thus, the treatment effects were most probably without pathological relevance. Another experiment with growing German Holstein bulls fed rations with a DON concentration of approximately 2.2 mg/kg DM did not reveal adverse effects on ASAT and GLDH activity either (Dänicke et al. 2002).

Unspecific effects of BVDV-vaccination become obvious when the red blood count indices are considered. These effects might be related to immune-related impacts in some publications discussed as immunotoxic effects (Batista-Duharte et al. 2014; Zhu et al. 2014). These effects are based, amongst others, on stimulation of the pro-inflammatory NLRP3 pathway, including the induction of pro-inflammatory cytokines such as TNF-α spreading from the site of i.m. injection to the systemic circulation and consequently also to the liver where an acute phase reaction might be initiated. Under these conditions, many metabolic effects might occur (Batista-Duharte et al. 2014; Zhu et al. 2014; He et al. 2015).

Based on the laboratory findings of the red blood count, a microcytic normochromic condition can be diagnosed for BVDV-vaccinated bulls relative to their mock-treated counterparts since erythrocyte counts, hematocrit, and MCV were all significantly decreased, particularly at day 70 of experiment, while hemoglobin concentration and MCH remained statistically uninfluenced. These changes are quite similar to toxic aluminum effects on red blood count in laboratory animals characterized by a decrease in erythrocyte count, hematocrit, and hemoglobin concentration and an increase in MCH (Zaman et al. 1993). In interpreting the significant treatment effects, it has to be considered that red blood count traits were within the corresponding reference ranges which are given at 5–10 T/L for erythrocytes, 28–38% for hematocrit, 9–14 g/dL for hemoglobin, 11–17 pg for MCH, and 46–65 fL for MCV (Moritz 2014). Interestingly, a time delay of approximately 7 weeks between the last (booster) vaccination and the effects on hematocrit and erythrocyte counts was required in the present experiment with bulls while the MCV appeared to be influenced earlier (Fig. 2d). A regenerative component of the erythrocyte drop was not detected in the present experiment when red cell distribution width (RDW) is evaluated as an indicator for anisocytosis. Rather, the average RDW varied between 14.1 and 14.5% and is lower than the reference range of 16–20% (Roland et al. 2014) suggesting a higher degree of erythrocyte size-uniformity and a lower presence of precursor cells.

Besides the effects of BVDV vaccination on red blood count, there appeared to be an interaction between DON exposure and vaccination, particularly for hematocrit at day 70 of experiment. While BVDV-vaccinated bulls of the CON and FUS I group clearly showed a lower hematocrit compared to their mock-treated counterparts, this distinct difference disappeared or was less clear at higher DON exposures (groups FUS II and III) generally characterized by dose-related increases in hematocrit (Fig. 3a). The phenomenon of a DON-dose related increase in hematocrit was also observed in cows (Dänicke et al. 2017) and pigs (Prelusky et al. 1994) and was discussed as a myelotoxic or a haemo-concentrating effect induced by treatment-associated variations in water intake. However, both water intake and RDW remained uninfluenced by increasing DON exposure in the present study making myelotoxic or haemo-concentrating effects less probable. Viability of human erythrocytes was not influenced at high concentrations of 10 μM DON (Parent-Massin and Parchment 1998) while erythrocyte progenitor cells were shown to respond quite sensitive with a cytotoxic DON level of 0.25 μM and a no effect level (NOEL) of 0.075 μM (Froquet et al. 2001). These concentrations would correspond to 74 and 22 ng DON per milliliter, respectively. Although DON was detected only in traces in blood of bulls of the present study (Winkler et al. 2016), experiments with dairy cows suggest that such DON concentrations in systemic circulation are within the realms of possibility in the ruminating bovine (Keese et al. 2008b; Winkler et al. 2014). Despite these obvious in vitro myelotoxic effects of unmetabolized DON, no information is available on cytotoxic effects of de-DON on these progenitor cells. The increase in erythrocyte counts and hematocrit as observed in the present experiment would rather suggest stimulating effects of feeding the FUS-contaminated diets. It has been discussed that bone marrow effects cannot be excluded because of the co-presence of other *Fusarium* toxins, i.e., modified forms of DON and ZEN such as DON-3-glucoside (4426 μg/kg), 3- and 15-acetyl-DON (545 and 3657 μg/kg), and ZEN-4-sulfate (183 μg/kg) besides the free DON and ZEN and other toxins like butenolid (2714 μg/kg), aurofusarin (25,904 μg/kg), culmorin (13,625 μg/kg), and 15- and 5-OH-culmorin (2350 and 12,081 μg/kg) (Dänicke et al. 2017). While modified forms of DON and ZEN are systemically active in their free forms due to rumen metabolism (Dänicke and Brezina 2013), much less is known about bone marrow effects of other co-contaminating mycotoxins. In addition, discussed possible interfering effects of the FUS-associated increase in DM-intake have to be borne in mind in an overall interpretation of treatment effects.

The PCA revealed that DM intake formed a cluster with water intake, blood urea concentration, BHB, GLDH, GGT, cholesterol and others suggesting associations and supporting the discussed results of the variance analyses. Antibody titers to BVDV were less close situated to this cluster but appeared to be associated with some traits of granulocytes and red blood count supporting the discussed treatment effects. The treatment effects can even be more distinctively distilled when all 42 variables recorded for each bull are condensed (Fig. 5). With a few exceptions, all bulls of group CON were situated in the lower right quadrant. Moreover, the vaccinated animals of this group appeared to form a sub-cluster. FUS-fed animals formed another large cluster distantly situated from the CON group with bulls of groups FUS III and FUS I separating in distinctive sub-clusters while bulls of group FUS II were located in between. The effects of vaccination appeared to be less clear compared to the CON group. This visual integrative representation of all the data makes clear that treatments clearly influenced the metabolic, hematological, and immunological status of the animals.

## Conclusions

The total antibody response to BVDV-vaccination was influenced by oral exposure to *Fusarium* toxin-contaminated feed containing mainly DON in a non-linear manner which might also be due to linear and non-linear specific isotype responses. A dietary DON-concentration of 5.66 mg/kg DM was shown to result in the strongest total antibody response while a concentration of 8.31 mg/kg DM lowered total antibodies.

Based on total BVDV antibody response, DON concentrations higher than 5.66 mg/kg DM corresponding to a DON concentration of 5.0 mg/kg at a reference DM content of 88% have to be regarded as critical. This concentration matches the guidance value for critical dietary DON concentration of 5 mg/kg (88% DM) according to the European Commission recommendation (European Commission 2006).

Future studies should consider the detection of isotype-specific BVDV antibodies to clarify the nature of the DON exposure related non-linear response of total antibodies. Moreover, BVDV challenge experiments in combination with BVDV vaccination and DON exposure could be useful in examining the DON-related effects on BVDV-antibody protection to infection.

The marked effects of BVDV-vaccination on red blood count indices are probably due to the aluminum-containing adjuvant. These effects as well as the interactions with DON-exposure require further elucidation.

## Electronic supplementary material

Supplemental Table 1 (DOCX 23 kb)
