# Supplementary material for: Antibody response of growing German Holstein bulls to a vaccination against bovine viral diarrhea virus (BVDV) is influenced by Fusarium toxin exposure in a non-linear fashion
Source: Mycotoxin Res. 2018 Feb 7;34(2):123–39. doi: 10.1007/s12550-018-0307-4 (PMC5891545; doi:10.1007/s12550-018-0307-4)
Supplement: Supplementary file 1 — (DOCX 23 kb) [file 12550_2018_307_MOESM1_ESM.docx]

**Supplemental Table 1.** Arithmetic mean values and standard deviations of antibodies to BVDV and results of the microneutralization test (MNT) which was performed as confirmatory test at day 70 of experiment only

| Time [Experimental day] | Group^a^ | BVDV vaccination^b^ | n | Antibodies [S/N%] | |  | MNT [Titres, max-min] |
| --- | --- | --- | --- | --- | --- | --- | --- |
|  |  |  |  | Mean value | Standard deviation |  |  |
| 1 | CON | - | 8 | 86.5 | 2.9 |  | n.a. |
|  |  | + | 8 | 84.9 | 2.9 |  | n.a. |
|  | FUS I | - | 6 | 88.7 | 3.2 |  | n.a. |
|  |  | + | 8 | 81.9 | 5.0 |  | n.a. |
|  | FUS II | - | 8 | 80.0 | 10.8 |  | n.a. |
|  |  | + | 8 | 81.3 | 13.0 |  | n.a. |
|  | FUS III | - | 8 | 79.1 | 3.3 |  | n.a. |
|  |  | + | 7 | 81.0 | 6.2 |  | n.a. |
| 21 | CON | - | 8 | 88.6 | 5.9 |  | n.a. |
|  |  | + | 8 | 80.9 | 9.3 |  | n.a. |
|  | FUS I | - | 6 | 87.7 | 4.2 |  | n.a. |
|  |  | + | 8 | 79.5 | 4.2 |  | n.a. |
|  | FUS II | - | 8 | 80.4 | 14.3 |  | n.a. |
|  |  | + | 8 | 62.5 | 19.6 |  | n.a. |
|  | FUS III | - | 8 | 81.4 | 6.7 |  | n.a. |
|  |  | + | 7 | 82.1 | 4.5 |  | n.a. |
| 28 | CON | - | 8 | 88.0 | 4.8 |  | n.a. |
|  |  | + | 8 | 47.4 | 25.9 |  | n.a. |
|  | FUS I | - | 6 | 86.7 | 9.9 |  | n.a. |
|  |  | + | 8 | 65.9 | 16.4 |  | n.a. |
|  | FUS II | - | 8 | 82.5 | 13.3 |  | n.a. |
|  |  | + | 8 | 33.8 | 12.6 |  | n.a. |
|  | FUS III | - | 8 | 83.4 | 3.1 |  | n.a. |
|  |  | + | 7 | 64.9 | 25.0 |  | n.a. |
| 47 | CON | - | 8 | 94.3 | 4.4 |  | n.a. |
|  |  | + | 8 | 48.8 | 20.9 |  | n.a. |
|  | FUS I | - | 6 | 89.3 | 7.6 |  | n.a. |
|  |  | + | 8 | 52.5 | 16.2 |  | n.a. |
|  | FUS II | - | 8 | 79.3 | 16.3 |  | n.a. |
|  |  | + | 8 | 39.3 | 13.8 |  | n.a. |
|  | FUS III | - | 8 | 84.5 | 6.9 |  | n.a. |
|  |  | + | 7 | 60.4 | 19.2 |  | n.a. |
| 56 | CON | - | 8 | 87.8 | 6.9 |  | n.a. |
|  |  | + | 8 | 52.4 | 23.3 |  | n.a. |
|  | FUS I | - | 6 | 91.0 | 2.8 |  | n.a. |
|  |  | + | 8 | 53.9 | 16.3 |  | n.a. |
|  | FUS II | - | 8 | 81.8 | 12.9 |  | n.a. |
|  |  | + | 8 | 42.0 | 15.3 |  | n.a. |
|  | FUS III | - | 8 | 85.4 | 6.2 |  | n.a. |
|  |  | + | 7 | 60.7 | 19.5 |  | n.a. |
| 70 | CON | - | 8 | 82.1 | 11.9 |  | <1/5 |
|  |  | + | 8 | 50.8 | 21.8 |  | 1/453 - 1/36 |
|  | FUS I | - | 6 | 85.0 | 2.4 |  | <1/5 |
|  |  | + | 8 | 54.0 | 15.9 |  | 1/227 - 1/7 |
|  | FUS II | - | 8 | 80.1 | 8.5 |  | <1/5 |
|  |  | + | 8 | 39.6 | 15.7 |  | 1/113 - 1/18 |
|  | FUS III | - | 8 | 79.6 | 5.3 |  | <1/5 |
|  |  | + | 7 | 62.6 | 15.2 |  | 1/90 - 1/28 |

^a^ deoxynivalenol/zearalenone (mg/kg dry matter): CON, 0.36/0.08; FUS I, 3.01/0.28; FUS II, 5.66/0.48; FUS III, 8.31/0.69

^b^ “-“ bulls were not vaccinated; “+” bulls were vaccinated against BVDV (Bovilis BVD-MD, MSD Animal Health, Schwabenheim an der Selz, Germany) into cervical musculature at day 1 and 21 (boost) of experiment
